# Audit of the community-directed treatment with ivermectin (CDTI) for onchocerciasis and factors associated with adherence in three regions of Cameroon

**DOI:** 10.1186/s13071-018-2944-z

**Published:** 2018-06-19

**Authors:** Guy-Roger Kamga, Fanny N. Dissak-Delon, Hugues C. Nana-Djeunga, Benjamin D. Biholong, Stephen Mbigha Ghogomu, Jacob Souopgui, Joseph Kamgno, Annie Robert

**Affiliations:** 1 Ministry of Public Health, N°8, Rue 3038 quartier du Lac, Yaoundé, Cameroon; 2 Centre for Research on Filariasis and other Tropical Diseases (CRFilMT), P.O. Box 5797, Yaoundé, Cameroon; 3 Institut de Recherche Expérimentale et Clinique, Faculté de santé publique, Université catholique de Louvain, Clos Chapelle-aux-champs 30 bte B1.30.13, BE-1200 Brussels, Belgium; 4 Institute of Biology of Molecular Medicine, Rue des professeurs Jeener et Brachet 12 BE-6041 Gosselies, Université Libre de Bruxelles, Brussels, Belgium; 5 Parasitology and Ecology Laboratory, Faculty of Science, University of Yaoundé 1, PO Box 812, Yaoundé, Cameroon; 6 Molecular and Cell Biology Laboratory, Department of Biochemistry and Molecular Biology, University of Buea, P.O. Box 63, Buea, Cameroon; 7 Faculty of Medicine and Biomedical Sciences, University of Yaoundé 1, P.O. Box 1364, Yaoundé, Cameroon

**Keywords:** Onchocerciasis, Ivermectin, Persistence, Treatment coverage, Adherence, Cameroon

## Abstract

**Background:**

After more than 15 years of community-directed treatment with ivermectin (CDTI) in the Centre 1, Littoral 2 and West CDTI projects in Cameroon, the epidemiological evaluation conducted in 2011 revealed that onchocerciasis endemicity was still high in some communities. To investigate the potential reasons explaining this high endemicity, a cluster coverage survey was conducted in April-May 2015 in three health districts (HD), to assess the implementation of the CDTI, the 2014 therapeutic coverage and the five-year adherence to treatment. A two-stage cluster design was considered during analyses, with data weighted proportionally to age and gender distribution in the population.

**Results:**

In the three HDs, 69 community leaders, 762 heads of households, 83 community drug distributors (CDD) and 2942 household members were interviewed. The CDTI organization and the involvement of heads of households were in average weak, with 84.0% (95% CI: 81.2–86.4%) of them who had not participated in activities during the 2014 mass drug administration (MDA). On average, six of ten community leaders declared that the period of treatment was decided by the health personnel while the CDDs selection was made during a community meeting for only 43.4% of them. The 2014 weighted therapeutic coverage was 64.1% (95% CI: 56.8–70.9%), with no significant difference in the three HDs. The survey coverages were lower than the reported coverages with a significant difference varying from 14.1% to 22.0%. Among those aged 10 years and above, 57.8% (95% CI: 50.2–65.1%) declared having taken the treatment each time during the last five MDAs with no significant difference among HDs, while 9.8% (95% CI: 7.5–12.8%) declared that they had never taken the drug. In multivariate analysis, the most important factors associated with the five-year adherence to treatment were high involvement in CDTI and age (40+ years).

**Conclusions:**

Despite more than 15 years of CDTI, there was still weak community participation and ownership, a lower coverage than reported and an average five-year adherence in the surveyed HDs. The reinforcement of the community ownership by the Ministry of Public Health officials and the timely procurement of ivermectin as requested by the communities are some measures that should be implemented to improve the therapeutic coverage, adherence to treatment and hence achieve onchocerciasis elimination. Further anthropological and entomological studies would provide better insights into our understanding of the persistence of the disease in these three CDTI projects.

## Background

Onchocerciasis, better known as river blindness, is a debilitating insect-borne parasitic disease caused by *Onchocerca volvulus* and transmitted *via* the bites of blackflies of the genus *Simulium*. Clinical manifestations associated with this filarial infection include severe itching, disfiguring skin conditions, and visual impairment which can lead to permanent blindness. Onchocerciasis is a neglected tropical disease (NTD) that creates stigma, generates and perpetuates poverty and is an impediment to socioeconomic development. The disease is currently endemic in 31 African countries, in Yemen and in two localized foci of two Latin American countries (Brazil and Venezuela). The disease used to be endemic in six countries of the Americas, but control efforts led in the elimination of the disease in four of them (Columbia in 2013, Ecuador in 2014, Mexico in 2015 and Guatemala in 2016) [1]. About 99% of the estimated 198 million people living in areas where transmission of the parasite is likely to occur are in Africa [2]. From the latest estimates, the prevalence of infection is about 17 million, although the likelihood that this figure might be underestimated is high [3].

Ivermectin is currently the only known effective and safe drug used for mass treatments against onchocerciasis. Although highly effective against microfilariae, its macrofilaricidal activity is quite modest, and microfilariae release by adult worms resume just a few months after treatment. Thus, treatments must be repeated, for at least 12–15 years, to comply with the quite long reproductive lifespan of the adult worm [4, 5]. The community-directed treatment with ivermectin (CDTI), proposed by the World Health Organization (WHO) through the African Program for Onchocerciasis Control (APOC), has significantly improved ivermectin treatment coverage [6–8], leading to a quite successful control of onchocerciasis. Indeed, reports support improvements in the coverage, both geographical and therapeutic, as the total number of treatments delivered increased gradually to reach 132 million in 2016 in the African region [2].

Despite this effort in increasing drug coverage, the disease persists in some foci. Indeed, recent phase 1A surveys conducted in 2011 by WHO/APOC revealed onchocerciasis prevalence above 60% in some foci of the Centre 1, Littoral 2 and West CDTI projects in Cameroon [9]. The reasons for such persistence of the infection are yet to be elucidated, and it is not clear whether this is due to low treatment coverage, systematic non-adherence of a proportion of the population (so-called systematic non-compliers), high transmission of the infection or suboptimal response of the parasite to ivermectin. In the aforementioned CDTIs projects, the reported coverages were always above the 65% threshold required to control the disease [10]. However, no study has confirmed those coverages. Coverage rates in a community may not give the full picture of program success because there may be individuals or groups who systematically do not comply over the years, and thus contribute to the transmission of the disease. A study on the adherence with eight years of annual ivermectin treatment in Cameroon and Nigeria showed that only 42.9% of the participants took six to eight treatments [11]. It has been suggested that age, gender, the perception of the susceptibility to be infected by the disease, CDTI organization, and some socio-economic characteristics of the population can influence the adherence to treatments [11–15].

The objective of the present survey was to understand the reasons explaining the persistence of onchocerciasis in three CDTI projects (Centre 1, Littoral 2 and West) in Cameroon by assessing: (i) the implementation of the CDTI; (ii) the 2014 MDA coverage; and (iii) the factors influencing the adherence to the treatment in these CDTI projects.

## Methods

### Study area

**Fig. 1:**
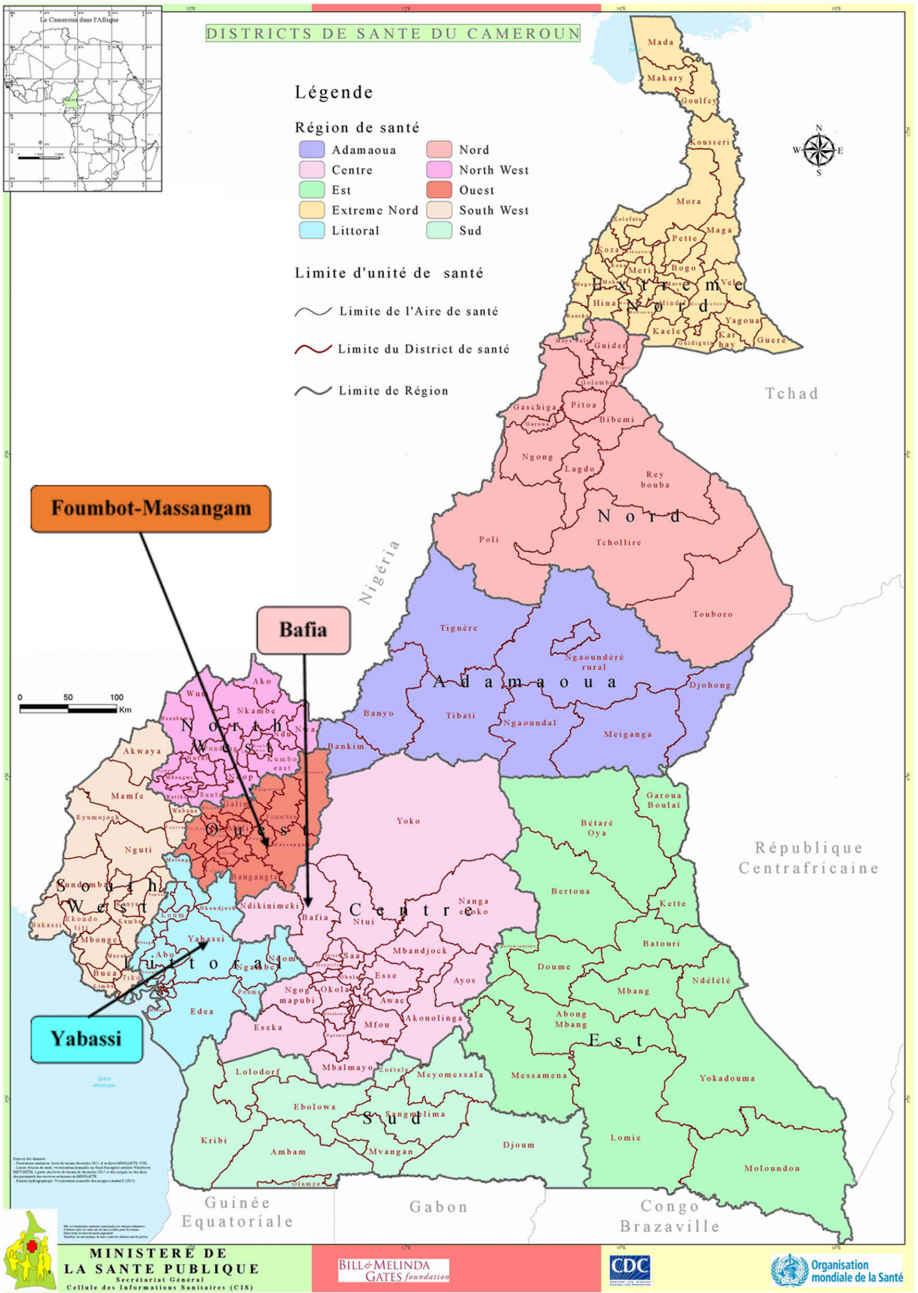
Map of Cameroon showing the surveyed health districts (source: 2016 © MINSANTE\SG\Cellule des Informations Sanitaires; https://www.dhis-minsante-cm.org/portal/ )

The present study was conducted in the Bafia (4°45'00"N, 11°14'00"E), Foumbot-Massangam (5°30'28"N, 10°37'57"E) and Yabassi (4°27'16"N, 9°57'56"E) health districts (HD), belonging to the Centre 1, West and Littoral 2 CDTI projects, respectively (Fig. 1).

The Bafia HD belongs to the Mbam and Inoubou Division (Centre Region), situated at 120 km north from Yaoundé, the political capital of Cameroon. In 2014, its population was estimated at 226,073 inhabitants, based on a census conducted by community drug distributors (CDD). The altitude of this Region varies from 1100 to 1300 m. It is a forest-savanna transition zone, irrigated by many fast-flowing rivers including the Sanaga and its tributaries, the main ones being the Mbam and the Noun rivers. The main activities of inhabitants are agriculture (mainly cocoa), fishing and sand mining.

The Foumbot-Massangam health district (currently split into two health districts) belongs to the Noun Division (West Region), at 25 km from Bafoussam, the regional capital. Census conducted by CDDs prior to the 2014 MDA revealed that its population was estimated at 132,847 inhabitants. It is a forest-savanna transition zone, irrigated by many fast-flowing rivers including the tributaries of the Sanaga in the east (Nkoup, Noun) that favor development of permanent blackflies’ breeding sites throughout the year. The relief of the region is mountainous with altitude varying from as low as 500 m in the Noun and Nkam valleys to 1000–2500 m in Western High Plateau. Agriculture is the main activity, involving at least 80% of inhabitants.

The Yabassi HD belongs to the Nkam Division (Littoral Region), at 100 km north-east from Douala, the economic capital of the country. According to the 2014 CDD census, its population was estimated at 21,459 inhabitants. The relief is undulating, showing an alternation of valleys and plains. The altitude of the Region varies between 10–800 m. This health district is also irrigated by many fast-flowing rivers comprising Nkam, Dibamba, Mabombé, Njanga, and Mahé which are favorable to blackfly breeding. The vegetation is mainly dense humid forest. Agriculture is the main activity, involving at least 60% of inhabitants.

### Study design

**Fig. 2:**
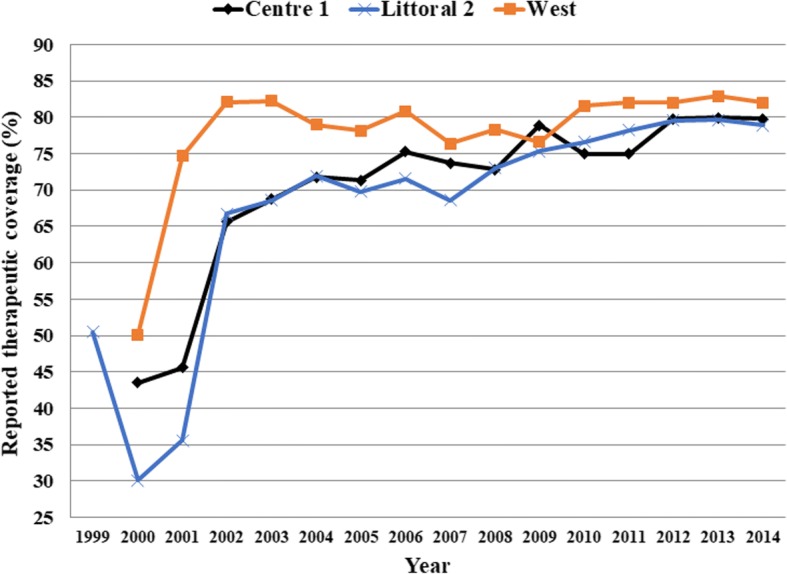
Therapeutic coverage of community-directed treatment with ivermectin in the Centre 1, Littoral 2 and West CDTI projects between 1999 and 2014

A cross-sectional survey was conducted in April and May 2015 in the health districts of Yabassi (Littoral Region), Bafia (Centre Region) and the former district of Foumbot-Massangam (West Region). Surveyed health districts were selected according to the results of the follow up epidemiologic evaluation carried out in 2011 by Katabarwa et al. [16] as well as the National Onchocerciasis Control Programme (unpublished report), revealing a persistence of high prevalence in those health districts, despite good reported therapeutic coverage [10]. Indeed, the three CDTI projects (Littoral 2, Centre 1 and West) reported sustained coverage above 70% during the last ten years. It should be noted that the formal CDTI started in 1999 in the Littoral 2, and in 2000 in the Centre 1 and the West projects, with a progressive enrollment of communities [10]. The reported therapeutic coverage achieved, calculated over the entire population, progressively increased from as low as 30% to above 80%, with some variations among projects as illustrated in Fig. 2. During the last five years/rounds of treatment, the treatment coverage was at least 75% in each CDTI project.

The selection of the clusters and the households was done following the World Health Organization guidelines for immunization coverage surveys [17, 18]. A total of 30 clusters (communities or villages) were selected in each health district according to the probability proportional to estimated size (PPES) strategy using the 2014 population figures obtained from the CDDs’ census.

### Data collection

In each cluster, eight households were randomly selected by a trained research assistant using the random walk method [19]. All the individuals living in a selected household, regardless of treatment eligibility, were interviewed about the last five MDA against onchocerciasis. Those who were present were asked to name all the current residents of the household and validate their age, sex, educational level, other socio-economic characteristics and their treatment status. Individual adherence to mass treatment was assessed by asking each participant if he/she swallowed ivermectin tablets during each of the previous five years (including the last CDTI-campaign). Ivermectin tablets were presented to the participants to make sure that the interview was targeting the right treatment. The treatment status of less than 10 years-old was given by their parents or caretakers. The members present were also asked about reasons any member did not take ivermectin during the 2014 distribution.

Once all the data were collected in a household, the research assistant attempted to compare interviewee declarations with the records in the CDDs registries. Unfortunately, it was not possible to cross-check the registries with the questionnaire data due to many reasons in all the surveyed districts: unavailable registries; lost registries destroyed by floods, fire; missing. Thus, from the survey data, treatment coverages were calculated and compared to 2014 treatment data reported by the health system. Data collection was done using a pre-tested questionnaire by the same research assistants in all HDs.

In each selected cluster, the community leader was interviewed, and one CDD was randomly chosen among the CDDs if they were more than one to work in the selected community using a specific questionnaire for each group.

#### Assessment of the conduct of the CDTI

The interviews of the heads of the households, the community leaders, and the community drug distributors were conducted using specific questionnaires for each group to assess the organization of the CDTI and their involvement. This, summarized as “involvement of the head of household”, used seven criteria to classify the overall CDTI process (“weak” for 0–3/7; “average” for 4–5/7; and “good” for 6–7/7 criteria achieved). These criteria and the reference answer (in parentheses) were: (i) the place of distribution (household); (ii) the actors responsible for the choice of the place of distribution (community members during a meeting); (iii) the involvement in the choice of the place of distribution (yes); (iv) the involvement in social mobilization activities (yes); (v) the actors responsible for the choice of CDDs (community members during a meeting); (vi) the involvement in the choice of CDDs (yes); and (vii) whether the interviewee attended health education sessions on onchocerciasis and its treatment in 2014 (yes). Each criterion received one point for the reference answer.

### Data analysis

All relevant data were recorded into a purpose-built Microsoft Access database and subsequently exported into STATA 13.1 for statistical analysis. As in a selected household, all the permanent residents were interviewed, the weighting was used to consider the difference in the population structure in terms of gender and age. The weighting was done according to the 2015 national demographic data projections [20]. Considering the cluster sampling, the design-adjusted Rao-Scott version of Pearson’s chi-square test (F) was used to compute in each health district, the 2014 treatment coverages, and the five-year adherence in the univariate analysis along with their 95% confidence intervals (CI) [21]. All variables having a *P*-value < 0.10 in univariate analysis were entered into a multiple logistic regression model to determine factors independently associated with the five-year adherence. The final model is reported with adjusted odds ratio (AOR) and 95% confidence interval (CI) for variables associated with the five-year adherence. *P*-values were corrected according to the Bonferroni method for multiple testing. We considered a *P*-value < 5% as indicative of a statistically significant result.

The following terms defined below by the WHO [22] and endorsed by Krentel et al. [23] and Shuford et al. [24], were calculated or used for analysis: therapeutic coverage, programmatic coverage, reported coverage, five-year adherence.

### Therapeutic (epidemiological) coverage

The proportion of individuals in the survey area who have swallowed ivermectin, out of the total population in the survey area, regardless of treatment eligibility.

#### Programmatic coverage

The proportion of individuals in the survey area who have swallowed ivermectin, out of the total population eligible (pregnant women, severely ill person and children less than five years-old are excluded from the total population) in the survey area.

#### Reported (administrative) coverage

The coverage calculated from data reported by all drug distributors, with census figures or drug distributor reports used to estimate the population denominator.

#### Adherence

Defined as “the extent to which a person’s behavior, taking medication, following a diet, and/or executing lifestyle changes, corresponds with agreed recommendations from a health care provider”. The five-year adherence was determined in individuals aged 10 years and above, who should have already taken at least five treatments, as the swallowing of ivermectin during each MDA of the five previous years (2010–2014).

## Results

**Fig. 3:**
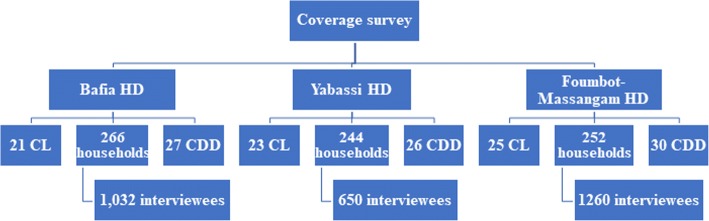
Flowchart indicating the distribution of participants interviewed. *Abbreviations*: CDD, community drug distributor; CL, community leader; HD, health district

In the three HD, 69 community leaders, 762 heads of households and 83 community drug distributors were interviewed (Fig. 3). The overall median number of inhabitants per household was 4 (Interquartile range (IQR): 3–6), corresponding to 4 (IQR: 3–6) in the Bafia HD, 5 (IQR: 3–6) in the Foumbot-Massangam HD, and 3 (IQR: 2–4), in the Yabassi HD. Women represented 52% of the 2942 individuals surveyed in the households. The mean age of participants was 25.4 ± 18.9 years, varying from 22.8 ± 17.7 in Foumbot-Massangam HD to 26.7 ± 20.0 in Bafia HD and 28.3 ± 18.8 in Yabassi HD (ANOVA: *F*_(2, 2906)_ = 21.96, *P <* 0.001).

### CDTI organization

**Fig. 4:**
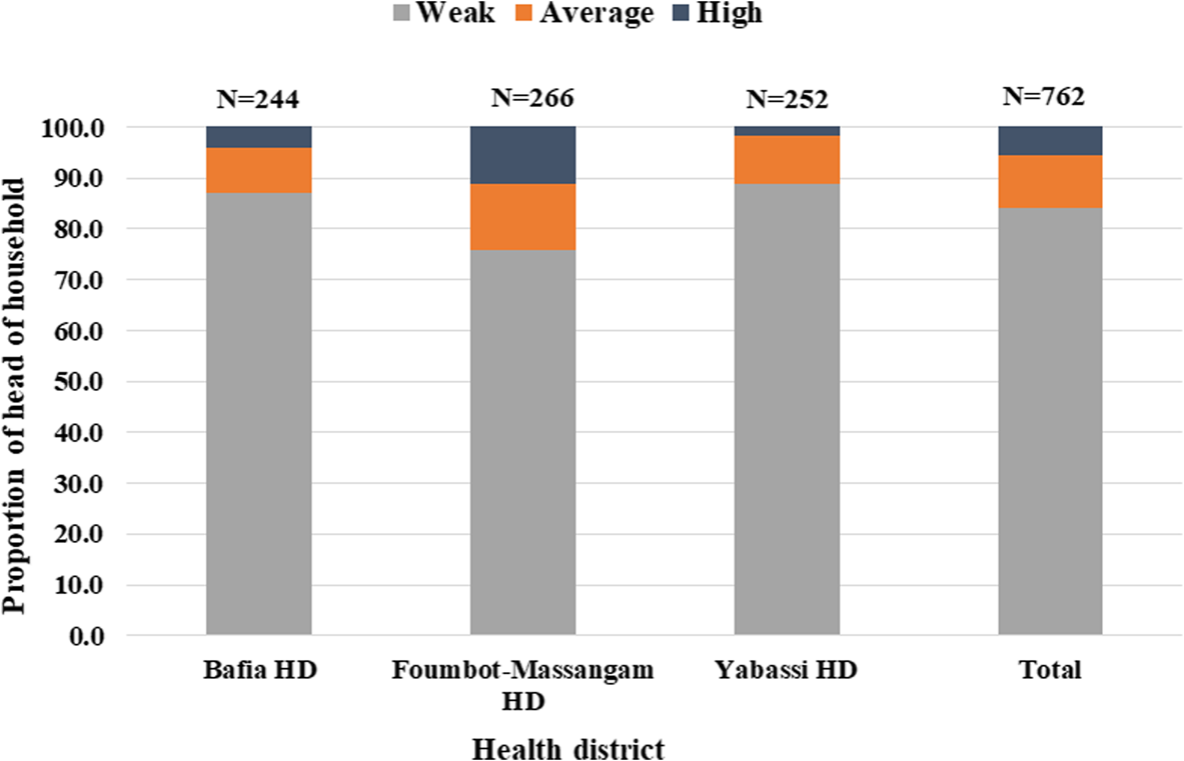
Organization and involvement of heads of household in the CDTI process

The organization of CDTI and the involvement of heads of the households were weak in the three HDs as in average 84.0% (95% CI: 81.2–86.4%) of them declared to not have participated in activities during the 2014 MDA campaign (Fig. 4). This weak participation was most important in the Yabassi HD 88.9% (95% CI: 84.3–92.3%) and in the Bafia HD 87.2% (95% CI: 82.6–90.7%) than in the Foumbot-Massangam HD 75.8% (95% CI: 70.1–80.7%) (*χ*^2^ = 27.2, *df* = 4, *P* < 0.001).

**Fig. 5:**
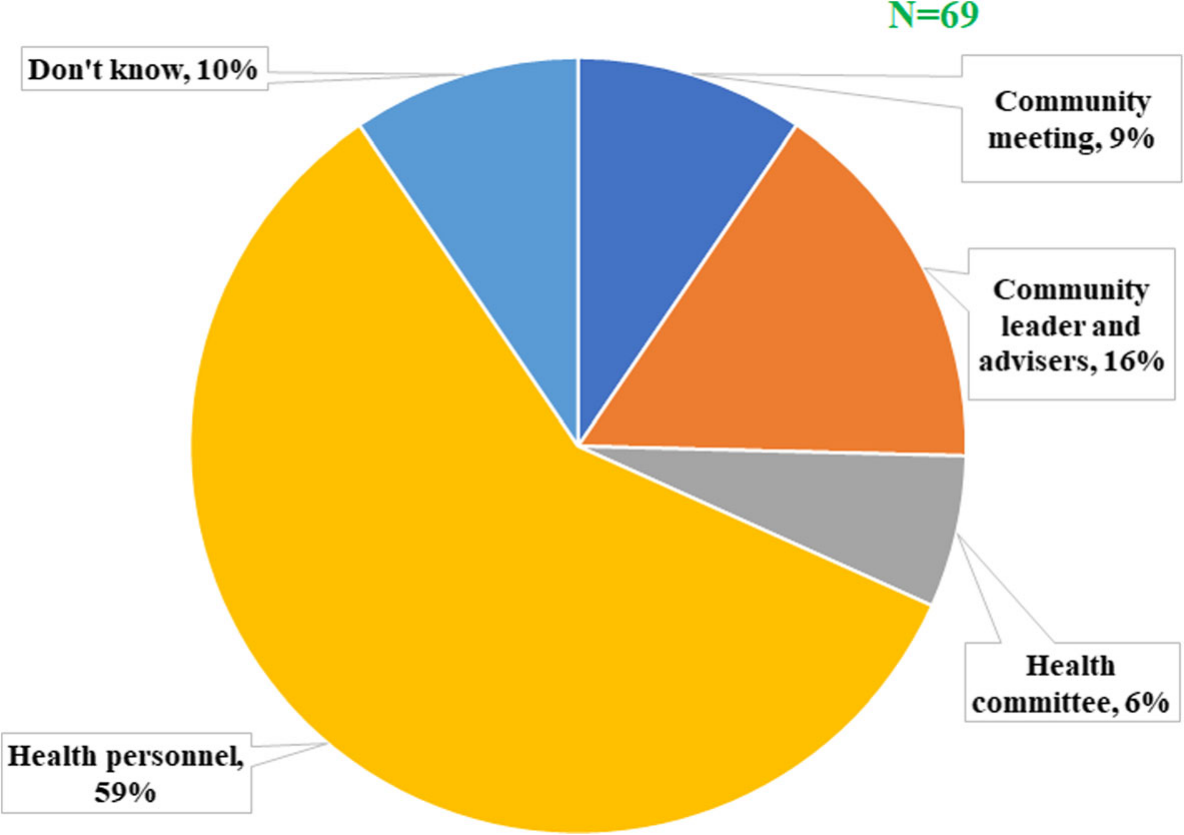
Actors responsible for the choice of the period of drug distribution according to community leaders

**Fig. 6:**
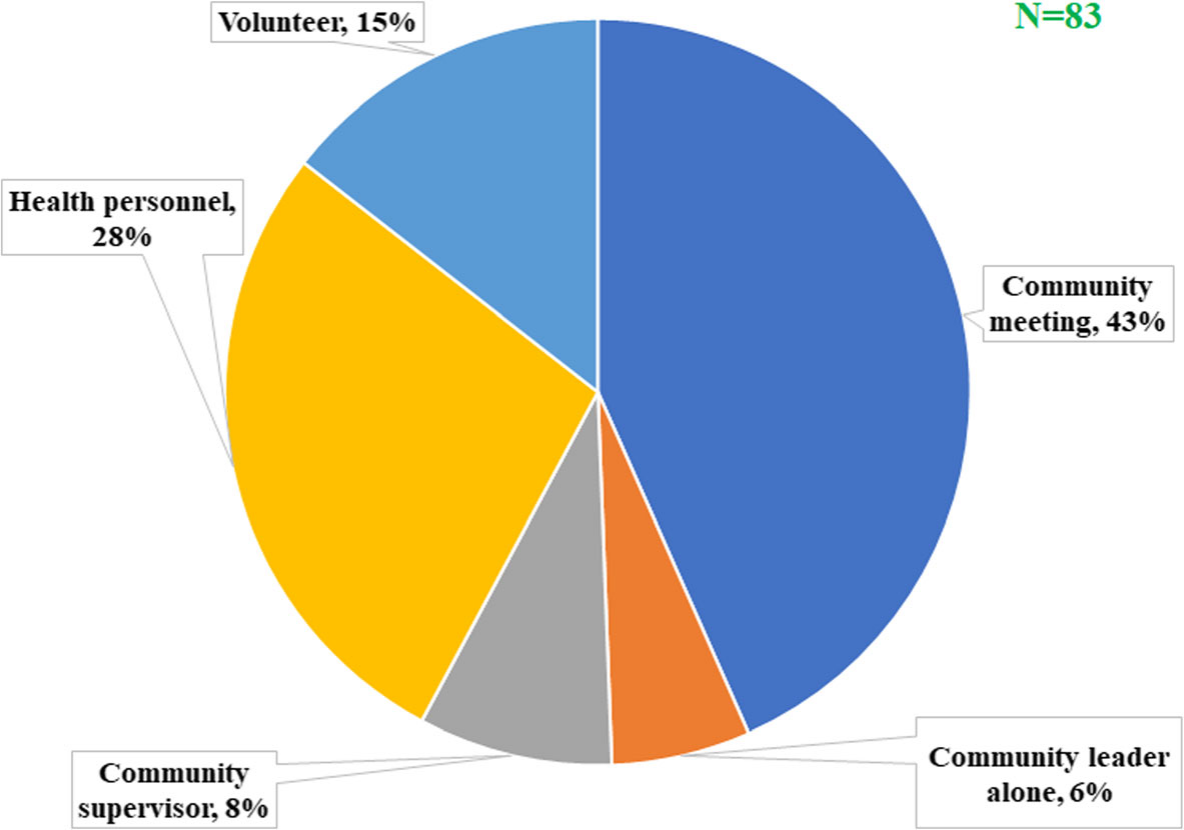
Actors involved in the choice of community drug distributors

The same observation about the community participation was made after interviewing the community leaders and the community drug distributors. On average, six out of ten community leaders declared that the period of mass drug administration was decided by the health personnel rather than the community members as recommended (Fig. 5), but no significant difference was found between HDs (*P* = 0.33). Almost the same proportion of individuals (57.1%) declared that the community was not involved in the selection of the CDDs. According to CDDs, their selection was made during a community meeting for only 43.4% of them, the other being mainly chosen by the health personnel (27.7%) or by self-selection (14.5%) (Fig. 6).

All the communities reported the late arrival of drugs usually in the rainy season during the farming period. At the district level, this late arrival was attributed to the late procurement by the National Onchocerciasis Control Programme (NOCP).

### Coverage of the 2014 MDA

**Fig. 7:**
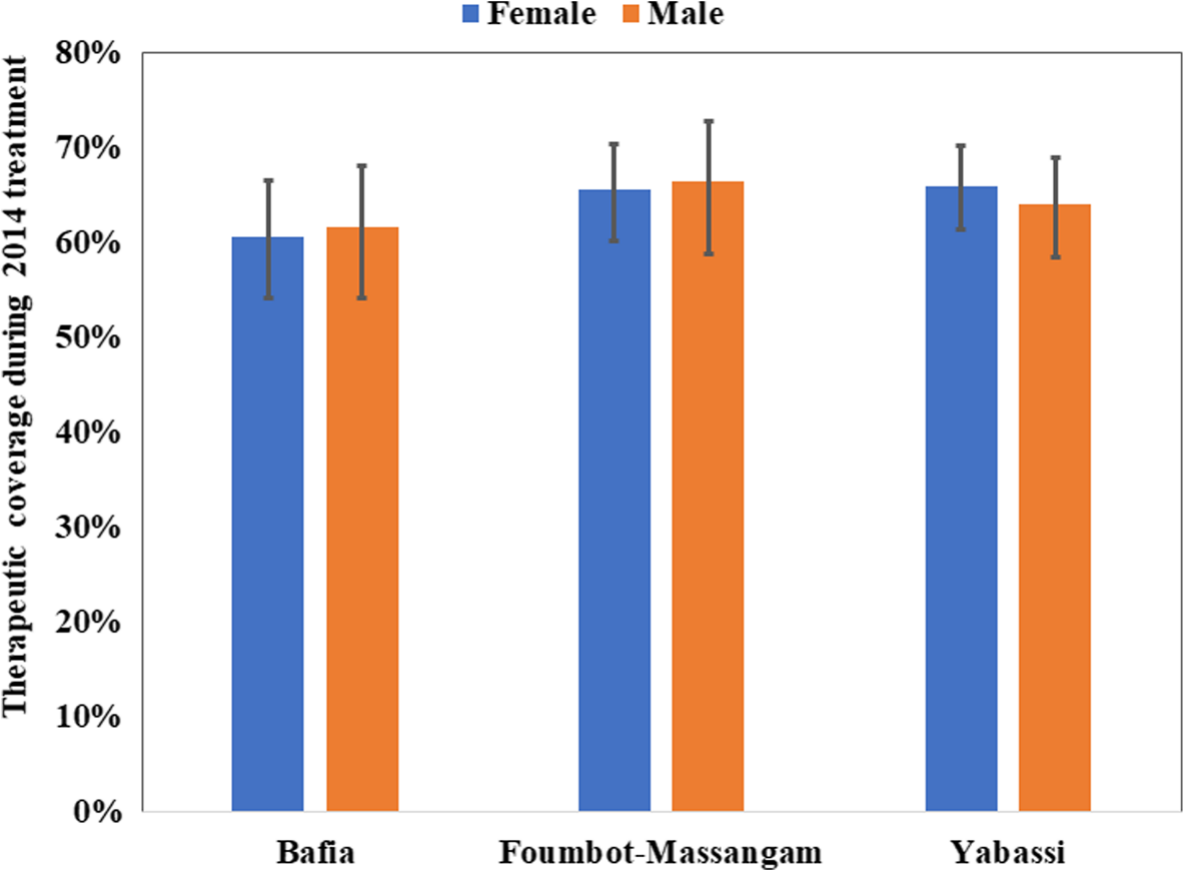
Therapeutic coverage during 2014 MDA by gender in the three districts (I-bars represent standard errors)

**Fig. 8:**
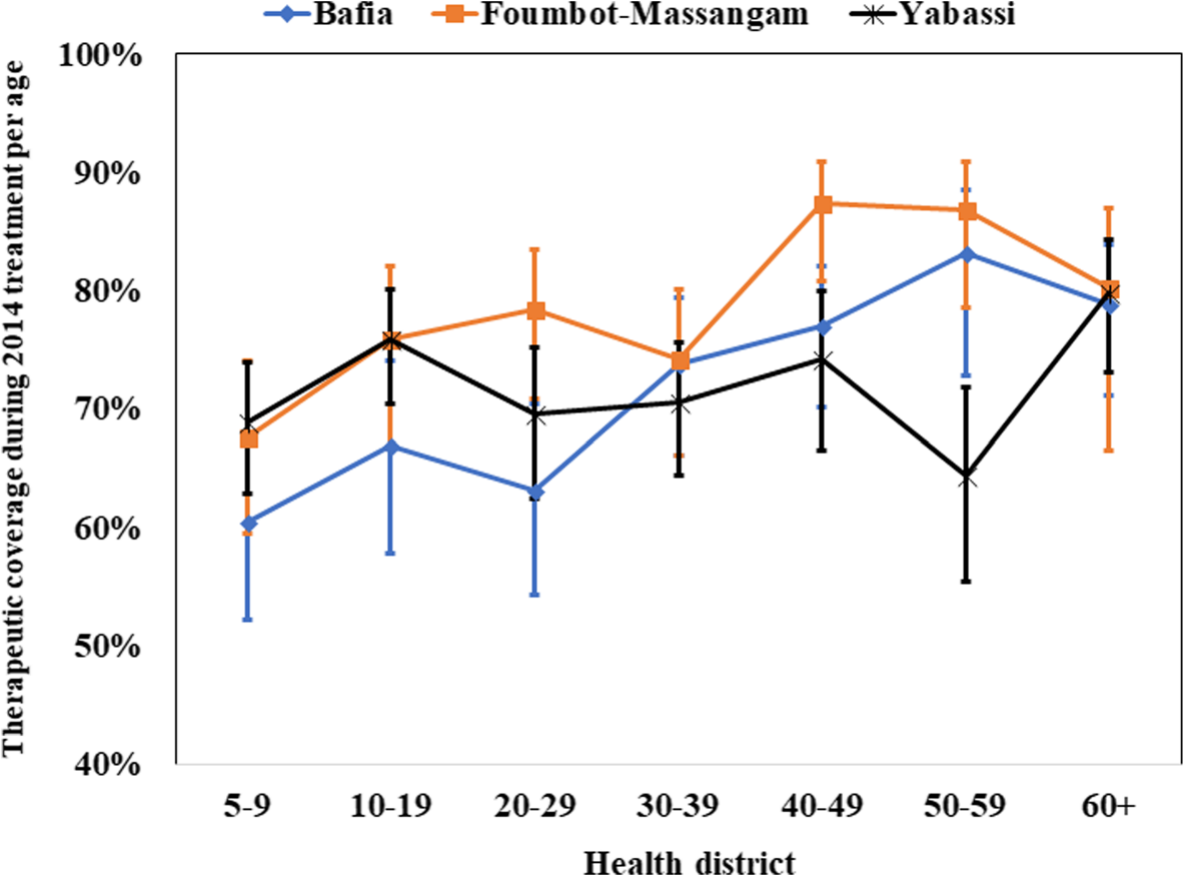
Therapeutic coverage during 2014 MDA by age in the three districts (I-bars represent standard errors)

**Fig. 9:**
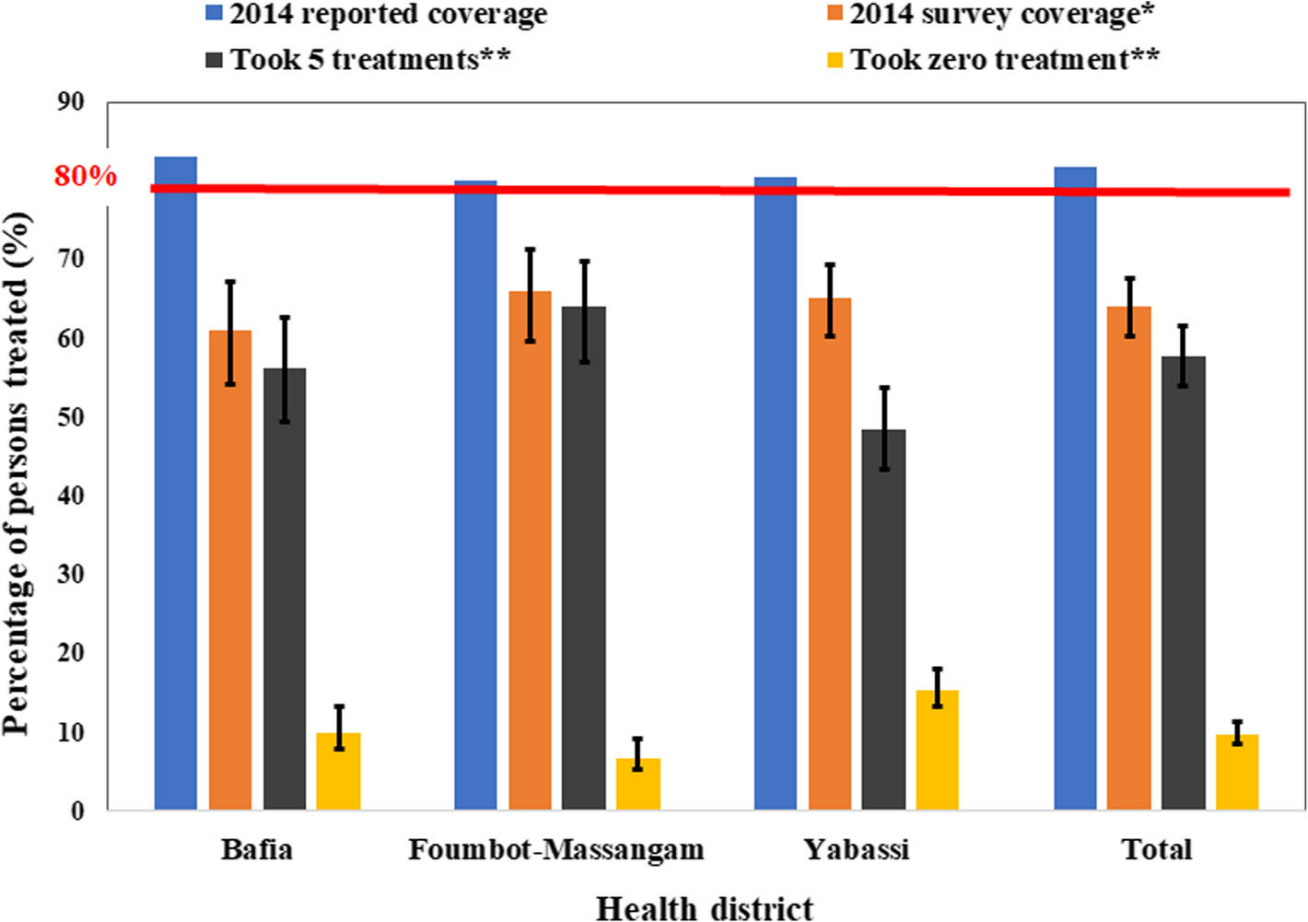
Therapeutic coverage during 2014 MDA and therapeutic adherence during the last five treatments (I-bars represent standard errors)

The 2014 overall weighted therapeutic coverage was 64.1% (95% CI: 56.8–70.9%) with almost no difference between women (64.0%; 95% CI: 57.3–70.2%) and men (64.3%; 95% CI: 55.7–72.0%) (*F*_(1, 89)_ = 0.02, *P* = 0.73) (Fig. 7). There was no significant difference between health districts with 61.1% (95% CI: 47.7–73.0%) in the Bafia HD, 65.1% (95% CI: 55.8–73.4%) in the Yabassi HD and 66.0% (95% CI: 53.6–76.6%) in the Foumbot-Massangam HD (*F*_(1.74, 154.48)_ *=* 0.23, *P* = 0.76). The weighted treatment coverage progressively increased with age, the highest coverages being observed among those aged 40 years and above (*F*_(2.46, 219.33)_ = 47.01, *P* < 0.001) (Fig. 8). This observation was similar in the three HDs. Private sector workers and their relatives, as well as those leaving in a household where the head was involved in the CDTI, had good therapeutic coverage. The detailed coverages in the three HDs according to participant characteristics are presented in Table 1. The 2014 coverages reported by the health system were higher than the observed during the survey. These differences varied from 14.1% in Foumbot-Massangam HD to 22.0% in Bafia HD (*χ*^2^ =558.22, *df* = 25, *P* < 0.001) (Fig. 9).

**Table 1:** The 2014 therapeutic coverage survey according to the characteristics of participants in each health district

		Bafia HD			Foumbot-Massangam HD			Yabassi HD		
		*n*	% (95% CI)	*P*	*n*	% (95% CI)	*P*	*n*	% (95% CI)	*P*
Gender				0.74			0.80			0.54
Female		531	60.6 (47.8–72.1)		646	65.6 (55.0–74.9)		337	66.0 (56.8–74.2)	
Male		496	61.6 (46.9–74.4)		594	66.5 (51.4–78.8)		305	64.0 (53.2–73.6)	
Age category (years)				**< 0.001**			**< 0.001**			**< 0.001**
0–4		82	Non-eligible		149	Non-eligible		57	Non-eligible	
5–9		152	60.5 (44.1–74.7)		194	67.6 (51.8–80.2)		55	68.9 (57.0–78.7)	
10–19		245	66.9 (49.2–80.9)		320	75.9 (57.8–87.9)		137	75.9 (65.2–84.1)	
20–29		160	63.1 (45.8–77.5)		193	78.5 (63.5–88.4)		123	69.6 (55.5–80.7)	
30–39		126	73.8 (58.6–84.8)		153	74.3 (58.3–85.7)		89	70.6 (58.5–80.4)	
40–49		105	77.1 (63.4–86.8)		103	87.4 (74.4–94.3)		74	74.3 (58.9–85.4)	
50–59		71	83.2 (62.8–93.6)		76	86.9 (70.7–94.8)		56	64.4 (46.8–78.9)	
60+		86	78.8 (63.7–88.7)		52	80.2 (53.3–93.5)		51	79.8 (66.7–88.7)	
Participants according to the profession of the head of household				0.21			**0.005**			0.14
Civil servant		99	51.1 (35.5–66.5)		134	74.3 (61.9–83.7)		67	75.4 (56.7–87.8)	
Private sector worker		27	72.0 (47.3–88.0)		37	93.4 (80.8–98.0)		19	84.3 (69.6–95.4)	
Farmer		611	65.4 (49.7–78.3)		603	63.5 (46.5–77.7)		240	59.2 (44.7–72.2)	
Trader		72	65.4 (40.8–83.8)		136	84.0 (77.3–88.9)		30	80.8 (66.9–89.8)	
Unskilled worker		52	61.2 (32.4–83.8)		106	47.8 (24.3–72.4)		89	70.9 (52.9–84.0)	
Jobless		163	46.7 (28.2–66.1)		219	62.1 (54.0–69.5)		190	61.1 (47.4–73.2)	
Participants according to the educational level of the head of household				0.28			0.09			0.59
Never attended school		83	76.6 (49.6–91.6)		83	52.8 (26.2–77.9)		45	52.7 (21.5–81.9)	
Primary school		422	60.4 (43.8–74.9)		572	69.0 (54.8–80.4)		243	64.1 (51.6–75.0)	
Secondary school		469	60.8 (45.1–74.6)		540	62.7 (50.0–73.8)		299	66.1 (56.5–74.5)	
University		51	48.2 (28.7–68.2)		45	93.7 (61.0–99.3)		52	74.0 (53.5–87.6)	
History of side effects during past treatments				0.23			0.97			**0.045**
No		935	63.5 (48.8–76.0)		1174	67.5 (54.1–78.5)		550	65.1 (54.3–74.5)	
Yes		59	51.1 (29.7–72.1)		32	67.9 (39.8–87.1)		72	80.4 (65.6–89.8)	
Perception of the risk to catch onchocerciasis				0.54			**0.005**			0.50
No		266	66.6 (47.9–81.3)		376	81.2 (73.9–86.8)		134	61.0 (47.2–73.2)	
Yes		757	59.4 (43.0–74.0)		859	59.7 (44.0–73.6)		508	66.2 (55.5–75.4)	
Involvement of parents in CDTI				**0.006**			**< 0.001**			**< 0.001**
High		51	93.0 (75.8–98.3)		109	94.4 (86.1–97.9)		18	88.6 (69.5–96.4)	
Average		91	66.0 (47.8–80.4)		193	84.3 (74.7–90.8)		79	88.5 (76.2–94.9)	
Low		885	58.9 (44.7–71.8)		938	59.6 (45.6–72.1)		545	61.0 (51.2–69.9)	
General therapeutic coverage		1027	61.1 (47.7–73.0)		1240	66.0 (53.6–76.6)		642	65.1 (55.8–73.4)	0.76*

*Abbreviations*: *HD* health district, *CDTI* community-directed treatment with ivermectin; *In bold*: *P*-value <5%; *P-value of the comparison between the three HDs

The 2014 overall weighted programmatic coverage was 74.4% (95% CI: 65.7–81.5%), with no difference between women (74.1%) and men (74.7%) (*F*_(1, 89)_ = 0.10, *P* = 0.76). These programmatic coverages varied from 69.1% (95% CI: 53.3–81.5%) in the Bafia HD to 74.4% (95% CI: 65.3–81.8%) in Yabassi HD, and 78.7% (95% CI: 62.4–89.1%) in the Foumbot-Massangam HD (*F*_(1.63, 145.38)_ = 0.66, *P* = 0.45).

**Fig. 10:**
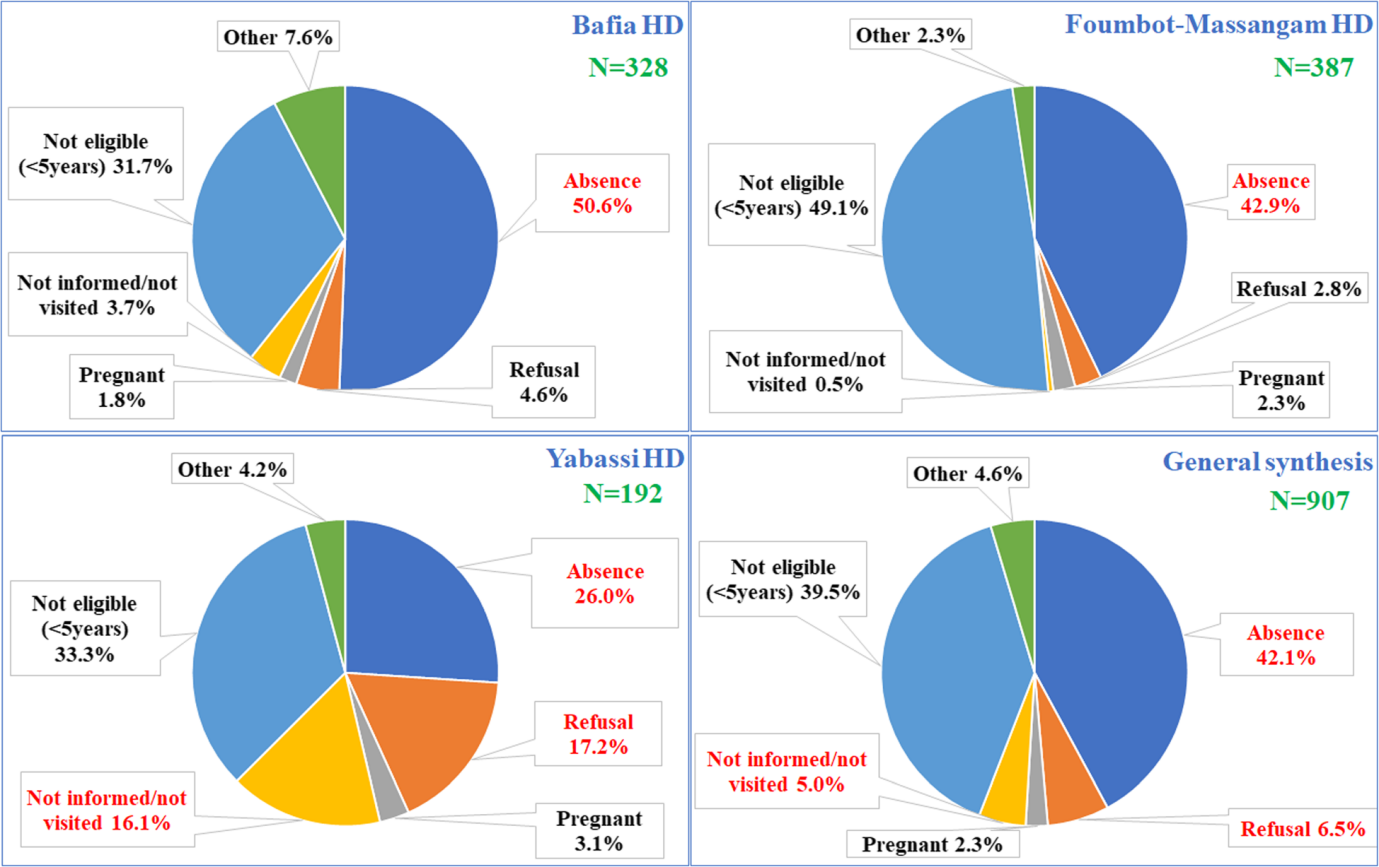
Reasons of non-treatment in the three health districts

Among those not treated in 2014, the reasons of non-treatment were assessed. In general, the main reasons were absence (42.1%) and refusal (6.5%). It should be mentioned that 5.0% of these untreated declared that they were not aware or not visited by the CDDs. The absence was the main reason evoked in the three HDs. However, in the Yabassi HD, refusals (17.2%) and the non-visits of the household (16.1%) were also important reasons. Non-eligible participants (less than 5 years old and pregnant women) at the time of the MDA represented 41.8% of untreated. Other reasons like shortage in ivermectin supply, payment requirement, belief of the inefficacy of ivermectin, and chronic illness were among reasons evoked by 4.6% of these untreated participants (Fig. 10).

### Adherence to ivermectin mass treatments

**Fig. 11:**
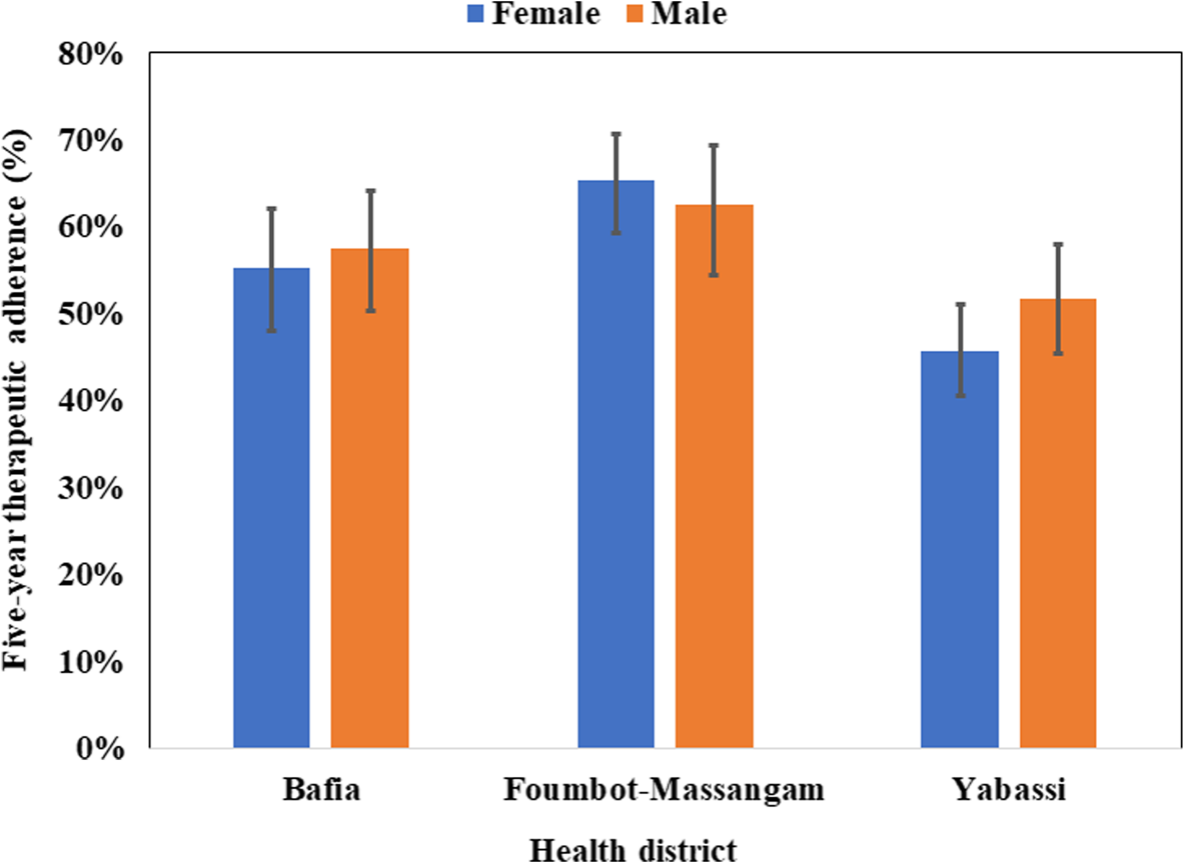
Five-year therapeutic adherence by gender in the three districts (I-bars represent standard errors)

**Fig. 12:**
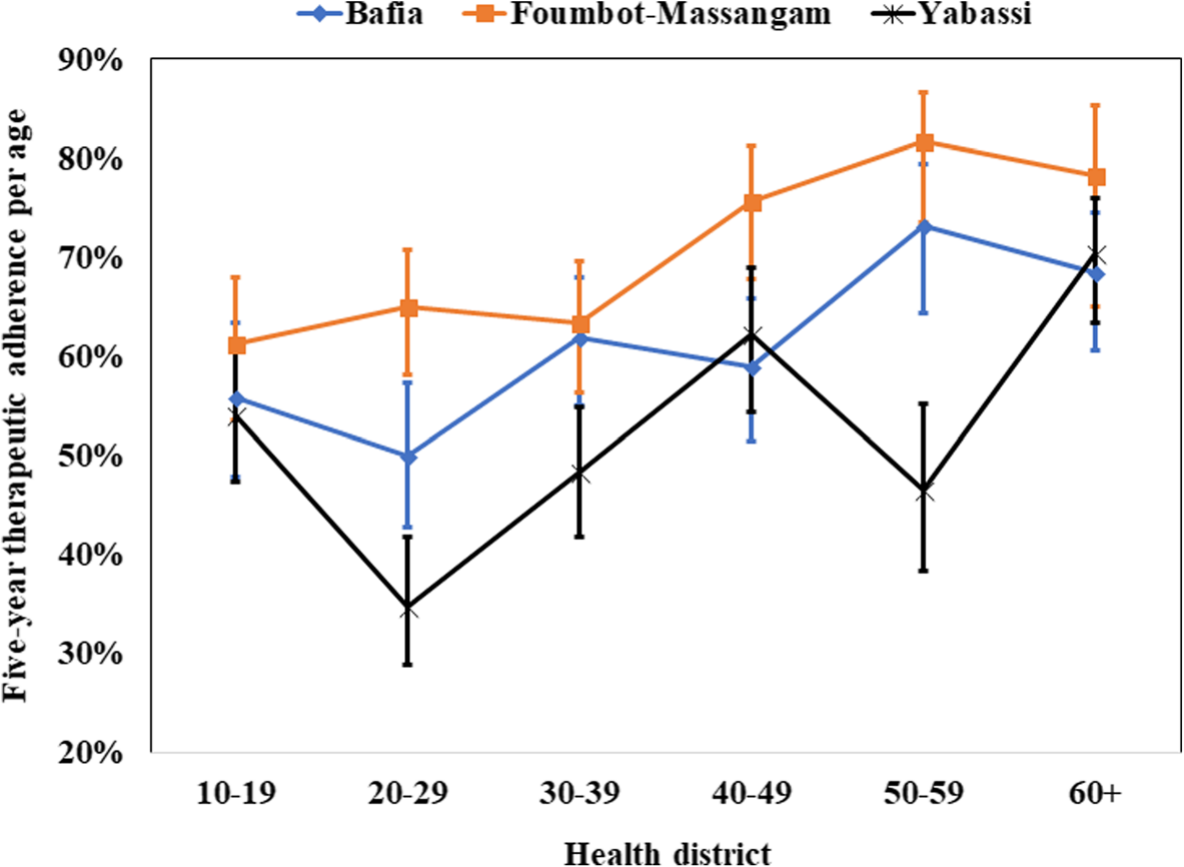
Five-year therapeutic adherence by age in the three districts (I-bars represent standard errors)

During our survey, 57.8% (95% CI: 50.2–65.1%) of the participants aged 10 years and above declared having taken ivermectin every time during the last five MDAs with almost no difference between women (57.3%; 95% CI: 49.9–64.5%) and men (58.4%; 95% CI: 49.7–66.6%) (*F*_(1, 89)_ = 0.18, *P* = 0.67) (Fig. 11). This proportion was higher in Foumbot-Massangam HD (64.0%; 95% CI: 50.6–75.6%) than in Bafia HD (56.4%; 95% CI: 42.9–68.9%), and Yabassi HD (48.5%; 95% CI: 38.4–58.7%), but the difference was not significant (*F*_(1.80, 160.39)_ *=* 1.45, *P* = 0.24). The weighted five-year adherence progressively increased with age, the highest adherence being observed among those aged 40 years and above (*F*_(3.17, 281.77)_ = 4.26, *P* = 0.005) (Fig. 12). This observation was similar in the three HDs.

Among these participants, 9.8% (95% CI: 7.5–12.8%) declared having not taken the drug during the last five MDAs. This proportion was higher in Yabassi HD (15.4%; 95% CI: 11.5–20.5%) than in Bafia HD (10.0%; 95% CI: 5.9–16.4%) and Foumbot-Massangam HD (6.8%; 95% CI: 3.8–11.6%). Among these non-adherent participants, 53% were females; 75% were less than 40 years-old; 98% had a weak involvement in the CDTI process and 67% were farmers or jobless. The characteristics of these participants are summarized in Table 2. The reasons of non-treatment given by persons not treated varied a lot: fear of the side effects, non-trust to the efficacy of ivermectin, non-trust to the CDDs, absence due to farming, distribution during an unsuitable period for the community, shortage in ivermectin, payment requirement, although it is known that the distribution is free of charge, temporary illness, as examples

**Table 2:** Characteristics of systematic non-adherents

		*n* = 222	%
Gender			
Female		118	53.1
Male		104	46.9
Age category (years)			
10–19		66	29.7
20–29		55	24.8
30–39		37	16.7
40–49		25	11.3
50–59		17	7.7
60+		9	4.0
Missing		13	–
Participants according to the profession of the head of household			
Civil servant		30	13.5
Private sector worker		2	0.9
Farmer		80	36.0
Trader		15	6.8
Unskilled worker		25	11.3
Jobless		68	30.6
Missing		2	–
Participants according to the educational level of head of household			
Never attended school		13	5.9
Primary school		87	39.2
Secondary school		104	46.8
University		17	7.7
Missing		1	–
History of side effects during pass treatments			
No		204	91.9
Yes		6	2.7
Missing		12	–
Perception of the risk to catch onchocerciasis			
No		62	27.9
Yes		160	72.1
Implication of parents in CDTI			
High		0	0
Average		5	2.3
Low		217	97.7

*Abbreviation*: *CDTI* community-directed treatment with ivermectin

The assessment of the determinants of the five-year adherence revealed that age (above 50 years), the profession of the heads of households (private sector worker), the perception of the risk to be infected and a high involvement in CDTI activities were the most favorable characteristics (Table 3). In the multivariate logistic regression, high involvement in the CDTI process (adjusted odds ratio, AOR: 19.01; 95% CI: 6.09–59.32) and age (40+ years, AOR: 1.58; 95% CI: 1.26–1.99) were the factors associated with the five-year adherence to treatments (Table 4).

**Table 3:** Five-year therapeutic adherence of persons 10 years and above in the surveyed districts

		Bafia HD			Foumbot-Massangam HD			Yabassi HD		
		*n*	% (95% CI)	*P*	*n*	% (95% CI)	*P*	*n*	% (95% CI)	*P*
Gender				0.58			0.43			0.25
Female		414	55.3 (41.3–68.5)		478	65.4 (53.4–75.7)		281	45.8 (35.6–56.3)	
Male		379	57.5 (43.5–70.4)		419	62.5 (46.7–76.0)		249	51.8 (39.4–64.0)	
Age category (years)				0.12			0.07			**0.004**
10–19		245	55.9 (40.1–70.7)		320	61.3 (46.2–74.5)		137	54.0 (40.9–66.6)	
20–29		160	50.0 (35.8–64.2)		193	65.0 (51.5–76.4)		123	34.7 (23.2–48.4)	
30–39		126	61.9 (48.3–73.9)		153	63.4 (49.4–75.4)		89	48.3 (35.5–61.3)	
40–49		105	59.0 (44.0–72.5)		103	75.7 (60.3–86.5)		74	62.2 (46.7–75.5)	
50–59		71	73.2 (56.0–85.4)		76	81.7 (65.7–91.2)		56	46.5 (30.4–63.5)	
60+		86	68.4 (53.2–80.5)		52	78.3 (52.4–92.2)		51	70.4 (56.7–81.2)	
Participants according to the profession of the head of household				**0.009**			0.09			0.48
Civil servant		82	39.3 (20.2–62.4)		98	75.2 (51.8–89.6)		56	58.5 (37.3–77.0)	
Private sector worker		21	91.6 (83.1–96.0)		32	89.4 (74.4–96.1)		17	47.1 (19.2–76.9)	
Farmer		444	64.9 (48.8–78.1)		442	62.3 (44.7–77.1)		189	50.6 (36.0–65.0)	
Trader		63	55.8 (33.4–76.0)		109	74.3 (55.5–87.0)		25	50.9 (30.3–71.2)	
Unskilled worker		45	55.6 (31.5–77.3)		74	40.5 (16.9–69.5)		77	59.9 (35.1–80.5)	
Jobless		136	34.9 (19.5–54.1)		138	61.7 (45.0–76.1)		161	37.8 (20.8–58.4)	
Participants according to the educational level of the head of household				0.50			0.13			0.47
Never attended school		68	70.2 (44.7–87.3)		63	54.4 (26.1–80.2)		37	49.2 (21.5–77.3)	
Primary school		322	57.5 (41.6–72.0)		412	67.0 (52.6–78.7)		201	45.4 (34.5–56.8)	
Secondary school		366	54.3 (38.2–69.5)		384	59.8 (44.5–73.3)		241	53.9 (42.7–64.7)	
University		36	44.9 (23.3–68.5)		38	92.6 (56.6–99.2)		50	34.4 (12.0–66.9)	
History of side effects during past treatments				**0.014**			0.93			0.66
No		735	57.5 (44.0–69.9)		859	64.5 (51.1–76.0)		451	48.7 (38.3–59.2)	
Yes		54	38.2 (23.4–55.5)		30	63.5 (34.2–85.3)		68	53.2 (32.6–72.8)	
Perception of the risk to catch onchocerciasis				0.70			0.19			**0.003**
No		213	53.2 (37.5–68.3)		282	71.9 (64.2–78.4)		114	27.3 (15.4–43.5)	
Yes		578	57.3 (40.9–72.2)		615	60.4 (42.4–75.9)		416	54.1 (43.7–64.2)	
Involvement of parents in CDTI				**0.005**			**< 0.001**			**< 0.001**
High		42	97.3 (86.9–99.5)		88	96.4 (77.1–99.5)		17	84.8 (75.6–90.9)	
Average		65	66.3 (39.9–85.3)		140	90.4 (84.2–94.4)		66	78.8 (54.2–92.1)	
Low		686	53.2 (39.0–66.9)		669	55.2 (40.8–68.7)		447	42.6 (32.4–53.5)	
Five-year adherence		793	56.4 (42.9–68.9)		897	64.0 (50.6–75.6)		530	48.5 (38.4–58.7)	0.24*

*Abbreviations*: *HD* health district, *CDTI* community-directed treatment with ivermectin; *In bold*: *P*-value <5%; *P-value of the comparison between the three HDs

**Table 4:** Predictive variables of the five-year adherence in multivariate logistic regression

Predictors variables	Adjusted odds ratio (95% CI)	*P*
Involvement in CDTI		< 0.001
High	19.01 (6.09–59.32)	
Average	4.38 (2.38–8.08)	
Low	1	
Age (years)		< 0.001
Less than 40	1	
40 and above	1.58 (1.26–1.99)	

*Abbreviation*: CDTI, community-directed treatment with ivermectin

## Discussion

The objective of the present survey was to investigate the reasons why onchocerciasis was persisting in the Centre 1, Littoral 2 and West CDTI projects by assessing: (i) the implementation of the CDTI; (ii) the 2014 MDA coverage; and (iii) the factors influencing the adherence to the treatment in three HDs selected in the targeted CDTI projects. This assessment was conducted before the publication of the 2016 new WHO field guide for implementation of coverage evaluation surveys for preventive chemotherapy [25]. However, our methodology was based on the well-known cluster survey method used by the Expanded Program on Immunization [17, 18].

In the three HDs, the interviews of the community leaders, the CDDs, and the heads of households revealed a weak community participation and ownership of the CDTI process. As recommended by the former APOC in the practical guide for the implementation of the CDTI, the selection of CDDs, the choice of the period of distribution among other processes should be made by the community members themselves [26]. In the surveyed HDs, these processes were mainly conducted by others (health personnel) rather than the community members. Consequently, the distribution period sometimes coincided with the farming activities, as declared by participants who were absent during the visit of the CDD. Furthermore, those who refused to take the treatment argued that the side-effects limited their ability to work while others mentioned that they did not trust the CDDs they had not chosen. As mentioned by many authors, a high community participation, ownership, good perception of the CDTI are associated with high coverages and a good adherence [24, 27–30], leading to a better control and a reduction of the transmission of onchocerciasis. The same observation was made in the three surveyed HDs as a high involvement in the CDTI had the highest predictive value of a good adherence. These findings emphasized the importance of the empowerment of the community participation by the Ministry of Health. If the community really decides on how and when to conduct the CDTI, its engagement will surely improve. Community leaders said that they did not see the importance of a better engagement if, at the end, community decisions will not be taken into account.

There was a great difference between the observed and the reported treatment coverage in 2014, ranging from 14% (Foumbot-Massangam HD) to 22% (Bafia HD). This difference can be attributed to many factors: an incomplete census leading to an underestimation of the total population, a misreporting of persons treated either voluntary or unintentionally, the refusal to treat some community members by the CDD due to interpersonal conflict, the demotivation of CDDs with impact on the quality of their work, as examples.

Unfortunately, the CDDs registries were not available to check the concordance of the data. However, we reported the same lower coverages in different settings in the same HDs [31–33]. Such over-reporting in the treatment coverage can bias or overestimate the success of CDTI thereby inhibiting all actions that can help improve the situation such as the reinforcement of the community empowerment and CDTI ownership. As mentioned earlier, the involvement of community leaders and heads of households in the CDTI also had a great effect on the treatment. In general, the coverages increased with age in the three HDs, as it is likely that the oldest who witnessed the consequences of the disease when its burden was high, before the start of control measures were therefore aware of the importance of treatment. Even though their number was low, household members from private sector had a better coverage. Private sector worker was defined as someone not paid by the government, but who received a salary at the end of the month from his employer. Due to the employment precarity and the scarcity of a stable paid work, private sector workers usually do anything possible to stay healthy and preserve their job. This is probably the reason motivating them to take the treatment and invite their relatives to do the same.

The effect of the educational level, the history of side effect during past treatments, as well as the perception of the risk to be infected by the disease on the adherence to treatment varied among the HDs.

Concerning the five-year adherence, 58% of participants declared having taken five treatments during the last five years while 10% of the participants did not take any treatment. Irregularly treated persons can constitute a reservoir for the disease transmission and be a threat to the disease elimination. Combined with other factors like the high transmission due to very high fly density and biting rates, this treatment irregularity can constitute an important reason explaining the persistence of the disease. It is worth mentioning that the better adherence recorded in the Foumbot-Massangam HD (64%), can explain at least, partially, the progress towards elimination observed in the West CDTI project [31]. The lower adherence observed in Yabassi and Bafia HD is likely to be among the main reasons explaining the persistence of the disease as recently reported [33]. With the observed adherence, the likelihood for the program to achieve elimination of the disease by 2025 is quite low. The factors independently associated with a good adherence in multivariate analysis were high parent involvement in the CDTI process (AOR: 19.01; 95% CI: 6.09–59.32) and age (40+ years, AOR: 1.58; 95% CI: 1.26–1.99).

The main limitation of our study was the fact that surveys were conducted six to nine months after the 2014 treatment, instead of being conducted one to three months after its completion to limit the recall bias. Data for the previous years were also subject to recall bias. However, we believe that our results regarding the therapeutic coverage and the adherence are reliable because ivermectin tablets are special, by both their physical presentation (small and white) and their delivery strategy. So, the likelihood to confuse them with other drugs or interventions is quite low. Brieger et al. [11] reported that 94% of participants were able to recall the number of times they took ivermectin during the previous eight years. Previous studies have also reported accurate recall of populations when compared to the data from CDTI collected in treatment registries [11, 34–36].

## Conclusions

The implementation of the community-directed treatment with ivermectin was not done as recommended in the surveyed health districts, with a weak community participation or ownership and a low five-year adherence despite more than 15 years since its inception. The reported therapeutic coverages were higher than those observed in the framework of this study and can, therefore, hide the real problems faced by the communities during the implementation of the CDTI, thus inhibiting the implementation of corrective measures. At the country level, the results of this study will help the National Onchocerciasis Control Programme to revise its strategies for the CDTI implementation (respect of the different steps, respect of the roles and responsibilities of the actors, timely procurement of ivermectin, effective supervision and data quality assessment). The findings of this survey were shared with the actors and used for a readjustment of the CDTI implementation. The reinforcement of the community ownership by the Ministry of Public Health officials should be implemented to improve both the coverage and the adherence and hence achieve onchocerciasis elimination. Further anthropological and entomological studies could also provide better insights into our understanding of the persistence of the disease in these three CDTI projects.

## Abbreviations

APOC: African Program for Onchocerciasis Control
ARES-CCD: Académie de Recherche et d’Enseignement Supérieur-Commission de la Coopération au Développement, Belgique
CDD: Community drug distributors
CDTI: Community-directed treatment with ivermectin
CI: Confidence interval
CL: Community leader
CRFilMT: Centre for Research on Filariasis and other Tropical Diseases
HD: Health district
IQR: Interquartile range
MDA: Mass drug administration
NOCP: National Onchocerciasis Control Program
WHO: World Health Organization
